# The digital dividend of the views on aging: how digital engagement influences subjective aging in Chinese older adults

**DOI:** 10.3389/fpubh.2026.1819089

**Published:** 2026-04-29

**Authors:** Yingtan Mu, Jiali Zhang, Qiuming Hu, Xiaofang Li

**Affiliations:** 1 Department of Social Security, Southwestern University of Finance and Economics, Chengdu, China; 2 Department of Social Work, School of Politics and Public Administration, Zhengzhou University, Postdoctoral Research Station of Public Administration, Zhengzhou University, Zhengzhou, China

**Keywords:** digital engagement, hierarchical growth curve model, perceived old age, subjective age, views on aging

## Abstract

**Background:**

Against the social backdrop of the simultaneous deepening of population aging and digital transformation, older adults’ subjective experience and evaluation of their own aging process have increasingly become core psychological elements influencing their physical and mental health as well as social integration.

**Methods:**

A nationally representative sample of older adults aged 60 and over from 3 waves (collected in 2018, 2020, and 2023) of The China Longitudinal Aging Social Survey was used in the analysis. Adopted as two-dimensional measures of Views on Aging (VoA) were subjective age and perceived old age, with their age trajectories among older adults and the cohort differences driven by digital engagement systematically examined via hierarchical growth curve models, and robustness checks conducted through propensity score matching.

**Results:**

Findings show that VoA of older adults become increasingly positive with age, with significant generational differentiation; digital engagement effectively boosts the positivity of their VoA (reflected in lower subjective age and higher perceived old age), while this positive effect diminishes marginally with age and displays distinct cohort differences.

**Conclusion:**

This study provides longitudinal empirical evidence on the reshaping effects of digital technologies on older adults’ VoA, and theoretical support for promoting active aging and building an age-friendly environment in the digital society.

## 1 Introduction

Population aging has become a fundamental and enduring trend in China’s social development, driven by the combined effects of persistently declining fertility rates and steadily rising life expectancy. The life expectancy of Chinese residents has risen substantially from 35 years in 1949 to 79 years in 2024^1^, resulting in a marked extension of the later stage of the life course. In this context, the conventional practice of defining old age as beginning at 60 is increasingly inadequate for capturing the heterogeneity and fluidity of older adults’ physical capacities, social roles, and psychological conditions. Consequently, examining how older adults subjectively experience and evaluate their own age and the aging process has emerged as an important avenue for understanding their social and psychological adaptation. Views on aging (VoA), namely individuals’ subjective appraisal of their age position and aging trajectory, not only reflect their meaning-making of growing old but have increasingly been recognized as a critical psychological predictor of aging attitudes, behavioral decisions, and social engagement.

Meanwhile, digitalization, as the core driving force of contemporary social development, has not only altered the patterns of information dissemination and social interaction but has also emerged as a crucial pathway for older adults to integrate into modern society and maintain social connections, releasing abundant digital dividends to all sectors of society. The term “digital dividends” refers to the economic and social benefits brought about by the popularization of internet technology, covering improvements in production efficiency, innovations in employment patterns, and the optimization of public services^2^. At the individual level, especially among older people, digital dividends are more prominently reflected in the well-being improvements generated through digital engagement. Specifically, by achieving digital access, digital usage, and digital inclusion, older adults gain convenience in information acquisition, social interaction, and daily life. This translates into enhanced well-being across multiple dimensions: physical health, mental state, social participation, and life satisfaction (1, 2). Digital engagement also serves as an important factor shaping older adults’ perceptions of their own age and judgments of the aging process, thereby influencing the formation and development of their VOA. According to the 56th *Statistical Report on Internet Development in China* released by the China Internet Network Information Center (CNNIC), the number of internet users aged 60 and above in China had reached 161 million by June 2025, accounting for 14.34% of the total online population. This trend reflects the rapid diffusion of digital technologies from younger to older age groups. Digital platforms not only provide older adults with opportunities for information acquisition, social interaction, and leisure activities but may also reshape their subjective perception by altering their understanding of social roles and experience of time (3). However, existing research on the relationship between digital engagement and VoA remains inconclusive. On the one hand, digital engagement may foster a more positive VoA by improving health status, enhancing self-efficacy, and strengthening social capital (4). On the other hand, it may also generate adverse consequences, such as information overload and internet addiction, thereby potentially intensifying age-related anxiety (5). Moreover, older adults at different life stages and from distinct cohorts exhibit substantial differences in their formative environments and social experiences, resulting in pronounced generational differentiation in age-related perceptions. This pattern is particularly salient in China, where traditional norms of respecting older adults coexist with modern youth-oriented values (6). Within this dual cultural context, older adults’ VoA are shaped not only by prevailing social norms but also reflect generational variations in social experiences, values, and lifestyles.

Building on this framework, the present study draws on three-wave longitudinal data from the China Longitudinal Aging Social Survey (CLASS) from 2018, 2020, and 2023. Hierarchical growth curve model is employed to examine the trajectories of older adults’ VoA, focusing on two dimensions: subjective age and perceived old age, while further assessing the role of digital engagement in shaping these trajectories. On this basis, the propensity score matching method is applied to more rigorously estimate the causal effects of digital engagement on VoA. By systematically elucidating the joint influences of intra-individual aging, cohort differences, and digital engagement within a unified longitudinal framework, this study provides empirical evidence to inform the development of age-friendly digital policies and the enhancement of subjective well-being among older adults, thereby offering important practical implications for promoting active aging.

## 2 Literature review

### 2.1 Measurement and research status of VoA

Views on aging (VoA) refer to individuals’ subjective experiences and evaluations of their own age status and the aging process (7). Existing research has mostly conducted theoretical interpretations and empirical analyses from the dual perspectives of social psychology and the life course. Among them, Age Identity Theory emphasizes individuals’ subjective definition of their own age identity, arguing that whether one is regarded as entering old age depends not only on chronological age but also on social comparison and role identification (8). From a developmental psychological perspective, Socioemotional Selectivity Theory proposes that with advancing age, individuals’ perception of future time becomes increasingly limited, which in turn raises the priority of affect-oriented goals (23), further shaping their cognitive and evaluative tendencies toward the aging process. In terms of specific measurement, VoA are generally conceptualized as a multidimensional construct, encompassing not only subjective perceptions of one’s own age but also social and psychological definitions of the boundary of old age (9). Subjective age and perceived old age are the most widely used indicators in relevant studies. Subjective age is typically operationalized using two primary approaches: multidimensional scales and single-item measures (10). The single-item approach generally asks respondents the question, “How old do you feel most of the time?” This item captures individuals’ overall cognitive positioning relative to their chronological age, reflecting a relatively stable age identity rather than a transient emotional or situational response (11). Empirical research suggests that developmental trajectories of subjective age are closely associated with well-being, health status, social motivation, and life engagement, while being jointly shaped by cultural contexts, social circumstances, and psychological states (12). In terms of variable construction, the relative difference between subjective age and chronological age allows for comparability across different age groups and is therefore more suitable for depicting developmental trends over the life course than the absolute difference (13). Accordingly, subjective age was operationalized as a percentage deviation score, calculated as 100 multiplied by the difference between subjective age and chronological age, divided by chronological age. Perceived old age, also referred to as the age threshold, refers to an individual’s judgment of when a person is considered old (8, 14). Typically measured by asking, “At what age do you think a person becomes old?” this construct reflects individuals’ psychological and social boundaries that demarcate entry into old age (15). This study simultaneously incorporates subjective age and perceived old age, which not only reduces the ambiguity associated with reliance on a single subjective evaluation but also enables mutual methodological validation, thereby strengthening the robustness of the research findings. By integrating both constructs, the present study mitigates the limitations inherent in single-indicator assessments while facilitating methodological triangulation, ultimately enhancing the robustness of the study’s conclusions.

So far, the primary focus of research has been on how VoA affect health status, psychological well-being, and social engagement. From a health-outcomes perspective, perceptions of aging are significantly linked to morbidity, chronic disease risk, and mortality. Stephan et al. (16) found that older adults who perceive themselves as younger experience slower declines in physical functioning and cognitive performance. Similarly, Westerhof et al. (22) reported that middle-aged and older individuals with fewer chronic conditions and lower levels of loneliness tend to report younger subjective ages. Beyond direct health outcomes, a more positive VoA can significantly buffer the psychological impact of negative life events (17). These relationships are partially mediated through social support networks and health-related behaviors (18).

### 2.2 Digital engagement and VoA

The increasing penetration of digital technologies among older adults has stimulated a growing body of research examining the relationship between digital engagement and VoA. Although a definitive consensus has yet to be reached, the majority of empirical evidence suggests a generally positive role of digital engagement, for which the Activity Theory of Aging provides core theoretical support. This theory posits that maintaining active social engagement and life participation can effectively slow the aging process and improve subjective aging experiences among older adults (24). As an extension and supplement to traditional social participation, digital participation is highly consistent with the “active aging” principle advocated by activity theory, thus constituting an important pathway for the older adults to construct positive views on aging in the new era. Existing empirical studies have offered substantial evidence for the above theoretical reasoning. Using data from the 2020 China Longitudinal Aging Social Survey, Kong and Zhu (4) demonstrated that digital engagement contributes to a younger subjective age by improving health status, enhancing self-efficacy, and strengthening social capital. Nevertheless, existing findings remain mixed and point to a more nuanced relationship. Excessive internet use, for instance, may reduce community involvement and offline social engagement (19). Elaborating on this complexity, Zhao et al. (20) identified a curvilinear association among Chinese adults aged 60 and above: moderate internet users reported a younger subjective age due to an enhanced sense of social value, whereas heavy users—whose participation in offline volunteer activities declined concurrently—perceived themselves as significantly older. Moreover, the digital divide introduces substantial heterogeneity in how VoA respond to technology use. Stratification by education, income, and geographic location generates unequal capacities for accessing digital resources, thereby shaping the extent to which older adults can derive cognitive and psychosocial benefits from digital engagement (21). In addition, challenges related to digital exclusion, regional disparities, and information verification independently exert negative influences on VoA outcomes (5).

Overall, existing researches have systematically demonstrated the significance of VoA in relation to health outcomes, psychological well-being, and social engagement, while also providing preliminary evidence that digital engagement may exert beneficial effects on VoA. However, several important gaps remain. First, prior studies have largely relied on cross-sectional designs, which limit the ability to examine the dynamic interplay between age-related changes and generational differentiation in aging self-perceptions. Second, digital engagement has often been treated as a homogeneous behavior, with insufficient attention to its potentially heterogeneous effects across different life stages and birth cohorts. Third, research on VoA has predominantly relied on single-indicator measures, neglecting the analytical advantages of integrating multiple dimensions such as subjective age and perceived old age. To address these limitations, the present study adopts a dual perspective of age trajectories and cohort differences analytical framework that combines subjective age and perceived old age to systematically examine how digital engagement influences VoA and whether these associations vary across generations. In doing so, this study aims to provide a more robust empirical foundation for understanding psychological aging trajectories within the context of an increasingly digitalized society.

## 3 Materials and methods

### 3.1 Data

We used microdata from the China Longitudinal Aging Social Survey conducted by Renmin University of China, a nationwide continuous survey on Chinese adults aged 60 and above covering their basic, health, socioeconomic, and information related to older adult care. Initiated in 2014 and completed five survey waves by 2023, the CLASS dataset includes observations from 2014, 2016, 2018, 2020, and 2023. To ensure data consistency and comparability across waves, this study utilizes data from the 2018, 2020, and 2023 waves. After excluding observations with missing key variables and those with only single-wave participation, a total of 18,536 valid samples were retained for analysis.

### 3.2 Measurements

This study adopts subjective age and perceived old age as two dependent variables to operationalize VoA among older adults, both of which are treated as continuous. Specifically, subjective age was measured using the CLASS questionnaire item, “How old do you feel most of the time?”, and was operationalized as a percentage deviation score calculated as 100 multiplied by the difference between subjective age and chronological age, divided by chronological age. Perceived old age was measured using the question, “At what age would you describe someone as old?”. Digital Engagement was measured using the question, “Do you use the internet?” Responses were dichotomized, with “never” coded as 0 and all other responses indicating internet use (e.g., daily or weekly use) coded as 1. In addition, gender, education, marital status, residential location, living arrangement, and the number of surviving children are included as control variables. For gender, males are coded as 1 and females as 0. Given the generally low educational attainment among older adults in China, educational level is categorized into two groups: primary school or below and junior high school or above. Self-rated health is measured on a five-point scale, ranging from 1 to 5 corresponding to “very healthy,” “relatively healthy,” “fair,” “relatively unhealthy,” and “very unhealthy,” respectively. Marital status is operationalized as a binary variable, where respondents who are married and living with a spouse are coded as 1, and all others as 0. Living arrangement is divided into four categories: living only with a spouse, living with children, living alone, and other arrangements. Residential location is a binary variable: respondents living in urban areas are coded as 1, while those residing in rural areas are coded as 0. The number of surviving children is treated as a discrete variable.

### 3.3 Analytical strategy

In this study, a hierarchical growth curve model is employed to examine how older adults’ VoA change with chronological age. This modeling strategy allows us to account for the dependence among repeated observations within individuals and to appropriately handle unbalanced panel data. The model incorporates chronological age, the quadratic term of age, cohort, the quadratic term of cohort, as well as the interaction between age and cohort. The specification is as follows.

#### 3.3.1 Level 1 model

Level 1 Model (Equation 1) captures the within-individual trajectory of VoA as a function of age, describing how an individual’s perception of aging evolves:


$$\begin{array}{l} y_{\mathrm{ti}}=\pi_{0i}+\pi_{1i}\mathrm{Ag}e_{\mathrm{ti}}+\pi_{2i}\mathrm{Ag}e_{\mathrm{ti}}^{2}+\sum_{j>2}\pi_{\mathrm{ji}}x_{\mathrm{jti}}+e_{\mathrm{ti}},\\ e_{\mathrm{ti}}∼N(0,\sigma^{2}) \end{array}$$ (1)

Where $y_{\mathrm{ti}}$ denotes the VoA of individual i at time t, $\mathrm{Ag}e_{\mathrm{ti}}$ and $\mathrm{Ag}e_{\mathrm{ti}}^{2}$ represent the chronological age of individual i at time t and its squared term, with age centered at the mean age of 70.344 years; $\pi_{0i}$ is the value of subjective age/perceived old age at the mean age; $\pi_{1i}$ and $\pi_{2i}$ are the linear and quadratic components of the trajectory slope corresponding to age, respectively; $x_{\mathrm{jti}}$ denotes all control variables; $\pi_{\mathrm{ji}}$ is the corresponding coefficient; and $e_{\mathrm{ti}}$ is the within-individual random error.

#### 3.3.2 Level 2 model


$$\begin{array}{l} \pi_{0i}=\beta_{00}+\beta_{01}\text{Cohor}t_{i}+\beta_{02}\text{Cohor}t_{i}^{2}+\beta_{03}\text{Interne}t_{i}\\ +\beta_{04}c_{i}I_{i}+\sum_{j>4}\beta_{0j}z_{\mathrm{ji}}+u_{0i} \end{array}$$ (2)

$$\begin{array}{l} \pi_{1i}=\beta_{10}+\beta_{11}\text{Cohor}t_{i}+\beta_{12}\text{Cohor}t_{i}^{2}+\beta_{13}\text{Interne}t_{i}\\ +\beta_{14}c_{i}I_{i}+\sum_{j>4}\beta_{1j}z_{\mathrm{ji}}+u_{1i} \end{array}$$ (3)

$$\begin{array}{l} \pi_{2i}=\beta_{20}+\beta_{21}\text{Cohor}t_{i}+\beta_{22}\text{Cohor}t_{i}^{2}+\beta_{23}\text{Interne}t_{i}\\ +\beta_{24}c_{i}I_{i}+\sum_{j>4}\beta_{2j}z_{\mathrm{ji}}+u_{2i} \end{array}$$ (4)

In the Level 2 model (Equations 2–4), $\beta_{00}$ is the average intercept of the age trajectory; $\beta_{10}$ is the average linear component of the age trajectory slope; $\beta_{20}$ is the average quadratic component of the age trajectory slope; $\text{Cohor}t_{i}$ denotes the cohort of individual i; $\text{Interne}t_{i}$ represents the status of the individual’s digital engagement; $c_{i}I_{i}$ is the interaction term between digital engagement and cohort for each individual; and $z_{\mathrm{ji}}$ denotes all time-invariant control variables.

## 4 Results

### 4.1 Descriptive analysis

Table 1 reports the descriptive statistics of the key variables. The analytical sample consists of 18,536 older adults, among whom 32.99% reported digital engagement, while 67.01% did not. This distribution is broadly consistent with the current level of digital technology adoption among the Chinese older adults. The mean subjective age was −4.355, indicating that, on average, respondents perceived themselves as 4.355% younger than their chronological age. The mean perceived old age was 70.374 years, substantially exceeding the conventional threshold of 60 years commonly used to define the onset of old age. Group comparisons reveal systematic differences between digitally engaged and non-engaged older adults. Individuals with digital engagement belonged to later birth cohorts, were younger in chronological age, and had significantly higher levels of educational attainment; they were also more likely to reside in urban areas. In contrast, non-engaged older adults were more likely to live in rural areas, had lower educational levels, and reported a larger number of surviving children. Notably, VoA differed consistently by digital engagement status: digitally engaged older adults reported a higher perceived age for the onset of old age and a younger subjective age relative to their actual age. These descriptive patterns provide preliminary empirical evidence of an association between digital engagement and VoA, which is examined more rigorously in the multivariate analyses that follow.

**Table 1:** Descriptive statistics.

Variable	Total (*N* = 18,536)		Non-digital engagement (*N* = 12,421)		Digital engagement (*N* = 6,115)	
	*N* (%) or Mean	SD	*N* (%) or Mean	SD	*N* (%) or Mean	SD
VoA						
Subjective age	−4.355	8.803	−3.929	9.132	−5.220	8.024
Perceived old age	70.374	11.317	69.613	11.371	71.918	11.047
Age	70.344	5.398	71.422	5.568	68.156	4.269
Cohort	1949.865	5.424	1948.534	5.541	1952.569	3.989
Gender						
Female	0.496	0.500	0.501	0.500	0.486	0.500
Male	0.504	0.500	0.499	0.500	0.514	0.500
Education level						
Primary school or below	0.613	0.487	0.734	0.442	0.366	0.482
Junior high school or above	0.387	0.487	0.266	0.442	0.634	0.482
Self-rated health						
Very healthy	0.158	0.125	0.019	0.138	0.009	0.092
Relatively healthy	0.107	0.310	0.118	0.322	0.086	0.280
Fair	0.371	0.483	0.403	0.490	0.306	0.461
Relatively unhealthy	0.435	0.496	0.397	0.489	0.513	0.500
Very unhealthy	0.071	0.258	0.063	0.243	0.087	0.283
Marital status						
No spouse	0.218	0.413	0.265	0.442	0.121	0.326
With spouse	0.782	0.413	0.735	0.442	0.879	0.326
Residential location						
Rural	0.426	0.494	0.517	0.500	0.241	0.428
Urban	0.574	0.494	0.483	0.500	0.759	0.428
Living arrangement						
Living with spouse only	0.592	0.491	0.545	0.498	0.687	0.464
Living with children	0.301	0.459	0.326	0.469	0.252	0.434
Living alone	0.091	0.288	0.110	0.312	0.055	0.227
Others	0.015	0.123	0.020	0.139	0.006	0.080
Number of living children	2.326	1.220	2.563	1.251	1.845	0.993

Compiled based on the 2018, 2020, and 2023 waves of CLASS data.

**Figure 1:**
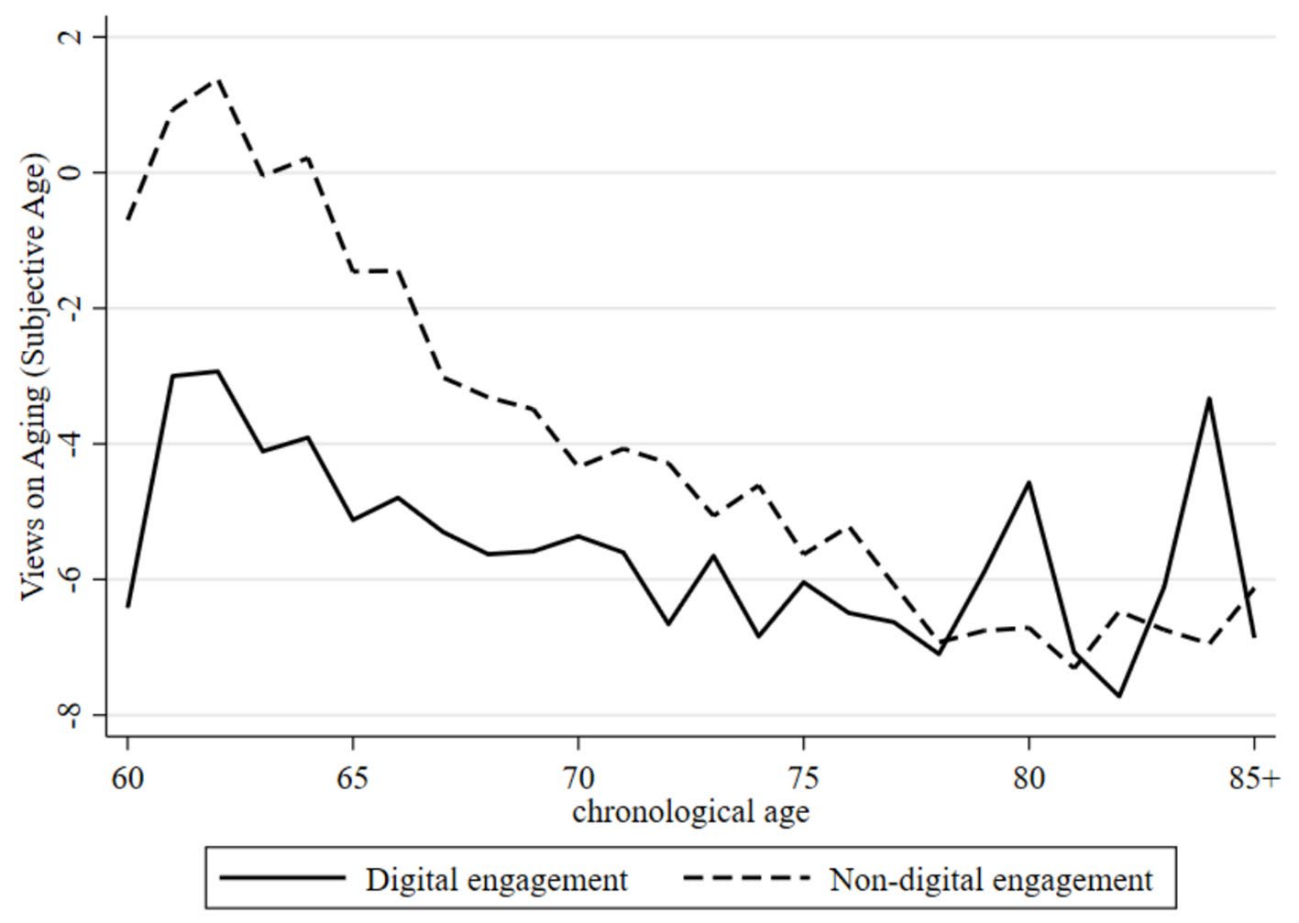
Trajectory of the VoA by age in the subjective age dimension. Compiled based on the 2018, 2020, and 2023 waves of CLASS data.

**Figure 2:**
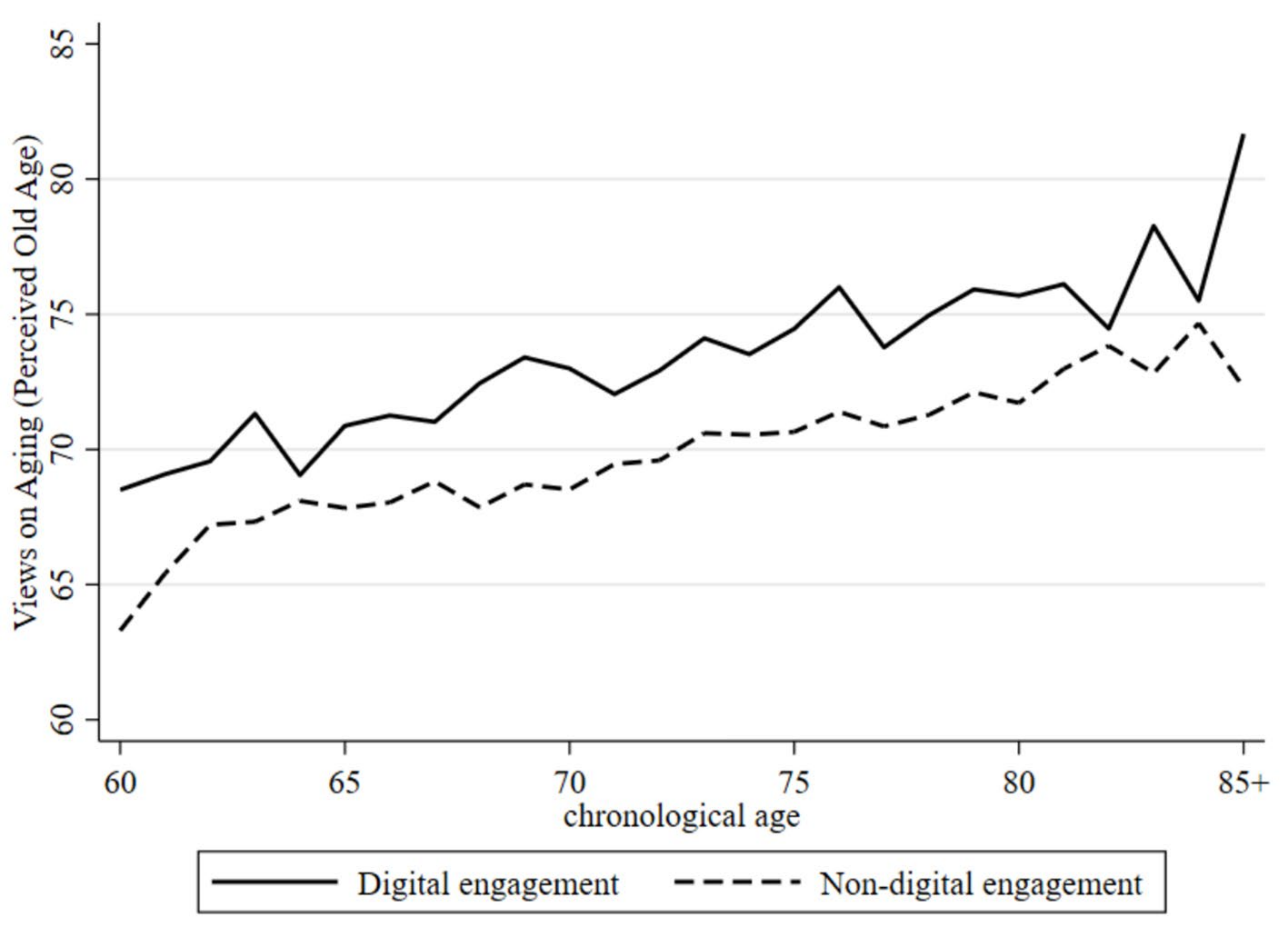
Trajectory of the VoA by age in the perceived old age dimension. Compiled based on the 2018, 2020, and 2023 waves of CLASS data.

To further illustrate the differential association between digital engagement and VoA, Figures 1, 2 depict the age-related trajectories of subjective age and perceived old age, respectively. As shown in Figure 1, digitally engaged older adults consistently reported younger subjective ages than their non-engaged counterparts. Although both groups exhibited a generally downward trend—indicating that individuals tended to feel progressively younger relative to their chronological age as they aged—the gap between the two groups gradually narrowed with advancing age. This pattern suggests that the association between digital engagement and subjective age may attenuate in later stages of old age. Figure 2 shows that digitally engaged older adults maintained consistently higher thresholds for perceived old age than those without digital engagement. While both groups displayed modest upward trends in perceived old age with increasing chronological age, the between-group differences persisted despite some fluctuation, lending further support to a positive association between digital engagement and delayed perceptions of aging.

### 4.2 Analysis of regression results

Growth curve modeling was employed to examine age trajectories and generational differentiations in VoA among Chinese older adults, as well as the role of digital engagement in shaping these patterns. Table 2 reports the hierarchical model estimates. Models 1 through 4 were sequentially specified, gradually incorporating core predictors, nonlinear age and cohort terms, cohort effects and their interactions, and finally a full set of sociodemographic and family structure covariates. Model fit improved consistently across specifications, with Model 4 yielding the lowest Akaike Information Criterion (AIC) and Bayesian Information Criterion (BIC) values. Accordingly, subsequent trajectory visualizations were based on the parameter estimates from Model 4.

**Table 2:** Growth curve model regression results for the VoA among older adults.

Variable	Subjective age				Perceived old age			
	Model 1	Model 2	Model 3	Model 4	Model 1	Model 2	Model 3	Model 4
Fixed-effect intercept model								
Intercept	−4.538^***^	−3.990^***^	−4.000^***^	−2.395^***^	69.14^***^	68.76^***^	68.66^***^	64.47^***^
	(0.141)	(0.159)	(0.162)	(0.622)	(0.186)	(0.210)	(0.213)	(0.790)
Age	−0.459^***^	−0.720^***^	−0.720^***^	−0.722^***^	0.271^***^	0.104^***^	0.103^***^	0.0938^***^
	(0.0171)	(0.0281)	(0.0281)	(0.0280)	(0.0207)	(0.0342)	(0.0342)	(0.0339)
Digital engagement (Ref.: Non-digital engagement)	−1.243^***^	−0.782^***^	−0.748^***^	−0.450^**^	3.801^***^	3.582^***^	3.956^***^	3.249^***^
	(0.143)	(0.151)	(0.184)	(0.188)	(0.180)	(0.191)	(0.235)	(0.238)
Age×digital engagement	0.208^***^	0.463^***^	0.460^***^	0.450^***^	0.215^***^	0.659^***^	0.624^***^	0.614^***^
	(0.0281)	(0.0504)	(0.0514)	(0.0512)	(0.0343)	(0.0616)	(0.0629)	(0.0623)
Cohort		−0.459^***^	−0.457^***^	−0.421^***^		−0.184^***^	−0.164^***^	−0.244^***^
		(0.0340)	(0.0345)	(0.0352)		(0.0432)	(0.0438)	(0.0447)
Cohort×digital engagement		0.345^***^	0.341^***^	0.332^***^		0.600^***^	0.549^***^	0.542^***^
		(0.0576)	(0.0593)	(0.0590)		(0.0718)	(0.0741)	(0.0734)
Age×cohort		−0.172^***^	−0.172^***^	−0.176^***^		0.0135	0.0118	−0.0183
		(0.0261)	(0.0261)	(0.0262)		(0.0324)	(0.0324)	(0.0322)
Digital engagement×age×cohort			0.00162	0.00136			0.0169^***^	0.0166^***^
			(0.00499)	(0.00496)			(0.00617)	(0.00612)
Quadratic model								
Age squared	0.0222^***^	−0.0581^***^	−0.0580^***^	−0.0594^***^	−0.00254	0.00658	0.00747	−0.00798
	(0.00197)	(0.0133)	(0.0133)	(0.0133)	(0.00236)	(0.0165)	(0.0165)	(0.0164)
Cohort squared		−0.110^***^	−0.110^***^	−0.111^***^		0.0106	0.0121	−0.00280
		(0.0135)	(0.0135)	(0.0135)		(0.0168)	(0.0168)	(0.0167)
Control variables								
Male (Ref: female)	−0.0388	−0.100	−0.101	0.0924	0.633^***^	0.676^***^	0.668^***^	0.552^**^
	(0.174)	(0.174)	(0.174)	(0.175)	(0.230)	(0.230)	(0.230)	(0.232)
Primary school or below (Ref: junior high school or above)				−0.987^***^				0.0615
				(0.195)				(0.258)
With spouse (Ref.: no spouse)				−0.862^***^				−0.103
				(0.229)				(0.290)
Self-rated health (Ref.: very healthy)								
Relatively healthy				0.543				2.283^***^
				(0.550)				(0.694)
Fair				0.0168				4.989^***^
				(0.528)				(0.666)
Relatively unhealthy				−0.619				5.921^***^
				(0.528)				(0.666)
Very unhealthy				−3.416^***^				6.202^***^
				(0.572)				(0.722)
Urban(Ref.: rural)				0.0418				1.485^***^
				(0.160)				(0.205)
Living with children (Ref.: living with spouse only)								
Living with children				−0.500^***^				−0.465^**^
				(0.155)				(0.195)
Living alone				−1.156^***^				−1.073^***^
				(0.288)				(0.362)
Others				−0.770				0.641
				(0.555)				(0.700)
Number of living children				−0.0413				−0.493^***^
				(0.0776)				(0.102)
Random effects: variance components								
sd (c_age)	0.598^***^	0.593^***^	0.593^***^	0.583^***^	0.557^***^	0.562^***^	0.563^***^	0.580^***^
	(0.024)	(0.023)	(0.023)	(0.023)	(0.037)	(0.036)	(0.036)	(0.035)
sd (_cons)	6.286^***^	6.259^***^	6.259^***^	6.201^***^	8.743^***^	8.719^***^	8.723^***^	8.645^***^
	(0.078)	(0.077)	(0.077)	(0.077)	(0.097)	(0.097)	(0.097)	(0.096)
sd (Residual)	5.022^***^	4.989^***^	4.989^***^	4.968^***^	6.233^***^	6.214^***^	6.210^***^	6.122^***^
	(0.039)	(0.038)	(0.038)	(0.038)	(0.048)	(0.048)	(0.048)	(0.047)
AIC	126119.8	125900.7	125902.6	125676.4	134653.3	134573.4	134567.9	134212.8
BIC	126190.3	126002.5	126012.2	125872.1	134723.7	134675.1	134677.4	134408.5
N	18,536	18,536	18,536	18,536	18,536	18,536	18,536	18,536

*p < 0.1; **p < 0.05; ***p < 0.01; values in parentheses indicate standard errors; age and cohort are centered.

Compiled based on the 2018, 2020, and 2023 waves of CLASS data.

Results for subjective age revealed that digital engagement was significantly associated with more positive VoA, as reflected in younger perceived ages relative to chronological age, however, this beneficial effect weakened with increasing chronological age, and significant cohort differences were also observed. Specifically, digitally engaged older adults reported significantly younger subjective ages than their non-engaged counterparts (*β* = −0.450, *p* < 0.01), confirming the salutary role of digital engagement in shaping self-perceptions of aging. Chronological age shows a significant negative main effect (*β* = −0.722, *p* < 0.01), suggesting that individuals tended to feel progressively younger than their actual age as they aged. Critically, the interaction between chronological age and digital engagement was positive and statistically significant (*β* = 0.450, *p* < 0.01), indicating diminishing marginal effects: although digital engagement was beneficial across all ages, its association with reductions in subjective age weakened among the oldest-old. This pattern aligns closely with the descriptive trajectories reported earlier. Cohort differences were also pronounced. The significant negative cohort main effect (*β* = −0.421, *p* < 0.01) suggests that younger generations reported more positive VoA. Moreover, the positive interaction between cohort and digital engagement (*β* = 0.332, *p* < 0.01) indicates that the association between digital engagement and subjective age varied across generations. The negative interaction between age and cohort (*β* = −0.176, *p* < 0.01) further implies that generational advantages in aging perceptions became more pronounced with increasing age. By contrast, the three-way interaction among digital engagement, age, and cohort did not reach statistical significance.

Turning to perceived old age, the results reveal a distinct yet complementary pattern. Regardless of digital engagement status, older adults tended to postpone the age threshold at which they defined someone as “old” as they themselves aged. However, this postponement was substantially more pronounced among digitally engaged individuals, with the divergence between groups widening over the life course. Unlike subjective age, which reflects personalized self-assessments, perceived old age captures normative judgments regarding the entry point into old age. Digitally engaged older adults reported significantly higher thresholds for perceived old age than their non-engaged counterparts (*β* = 3.249, *p* < 0.01), suggesting that digital engagement fosters more positive VoA by elevating the chronological standard at which old age is defined. Chronological age exerted a significant positive main effect (*β* = 0.094, *p* < 0.01), indicating that older participants consistently set higher age boundaries for old age. This pattern accords with social comparison theory, particularly the notion of downward comparison, whereby aging individuals recalibrate age norms to preserve positive self-evaluations. Crucially, the interaction between chronological age and digital engagement was positive and significant (*β* = 0.614, *p* < 0.01), demonstrating that, unlike subjective age, the beneficial association between digital engagement and perceived old age intensified rather than attenuated with advancing age. This amplification effect likely reflects enhanced access to information, social connectivity, and personal agency afforded by sustained digital engagement in later life. Cohort differences were again evident. The significant negative cohort main effect (*β* = −0.244, *p* < 0.01) indicates that younger cohorts maintained higher perceived thresholds for old age, while the positive interaction between cohort and digital engagement (*β* = 0.542, *p* < 0.01) suggests that the postponement of aging perceptions associated with digital engagement was especially pronounced among newer generations, consistent with their differential exposure to and familiarity with the digital environment.

**Figure 3:**
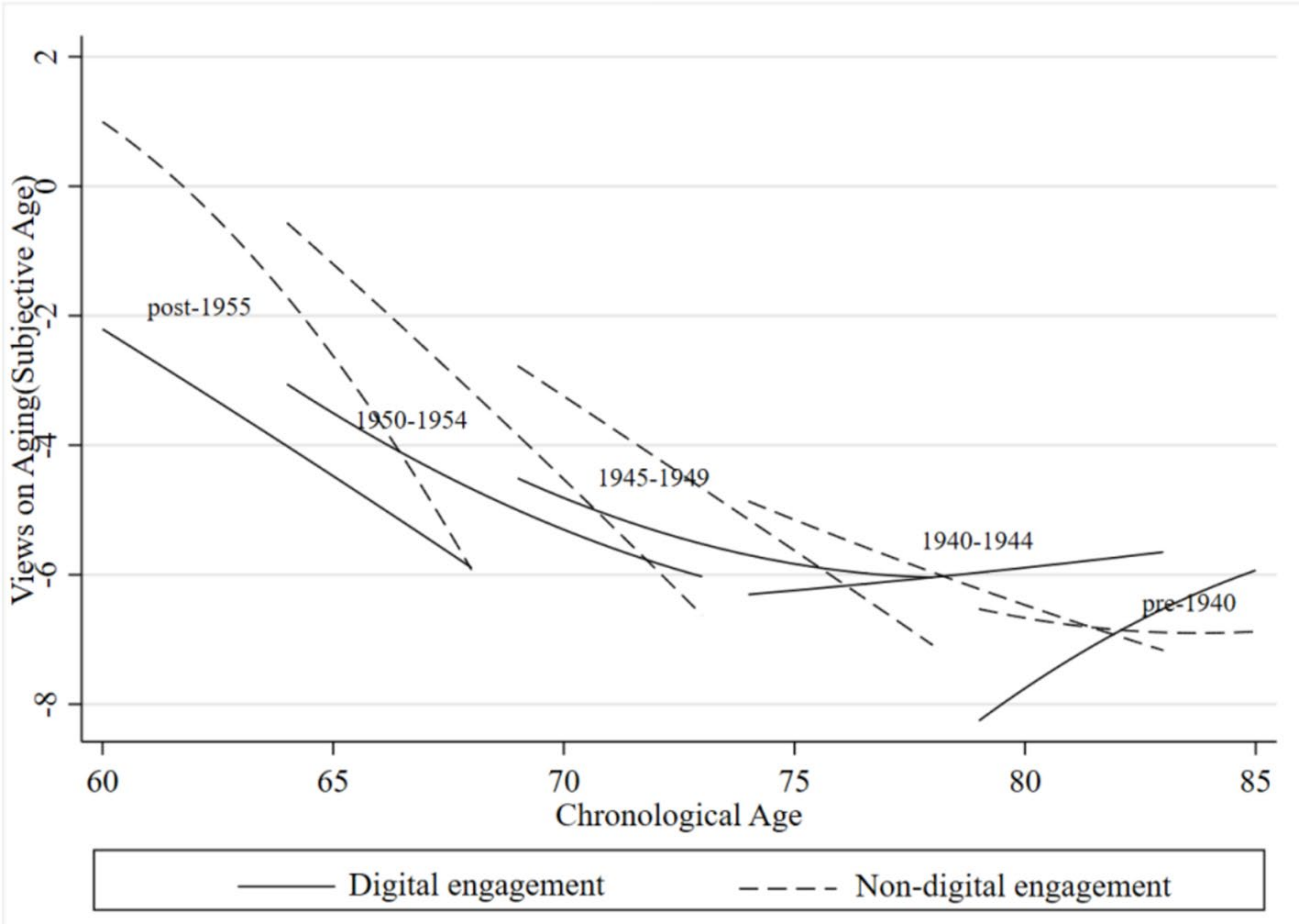
Age trajectories of subjective age for older adults across generations. Compiled based on the 2018, 2020, and 2023 waves of CLASS data.

**Figure 4:**
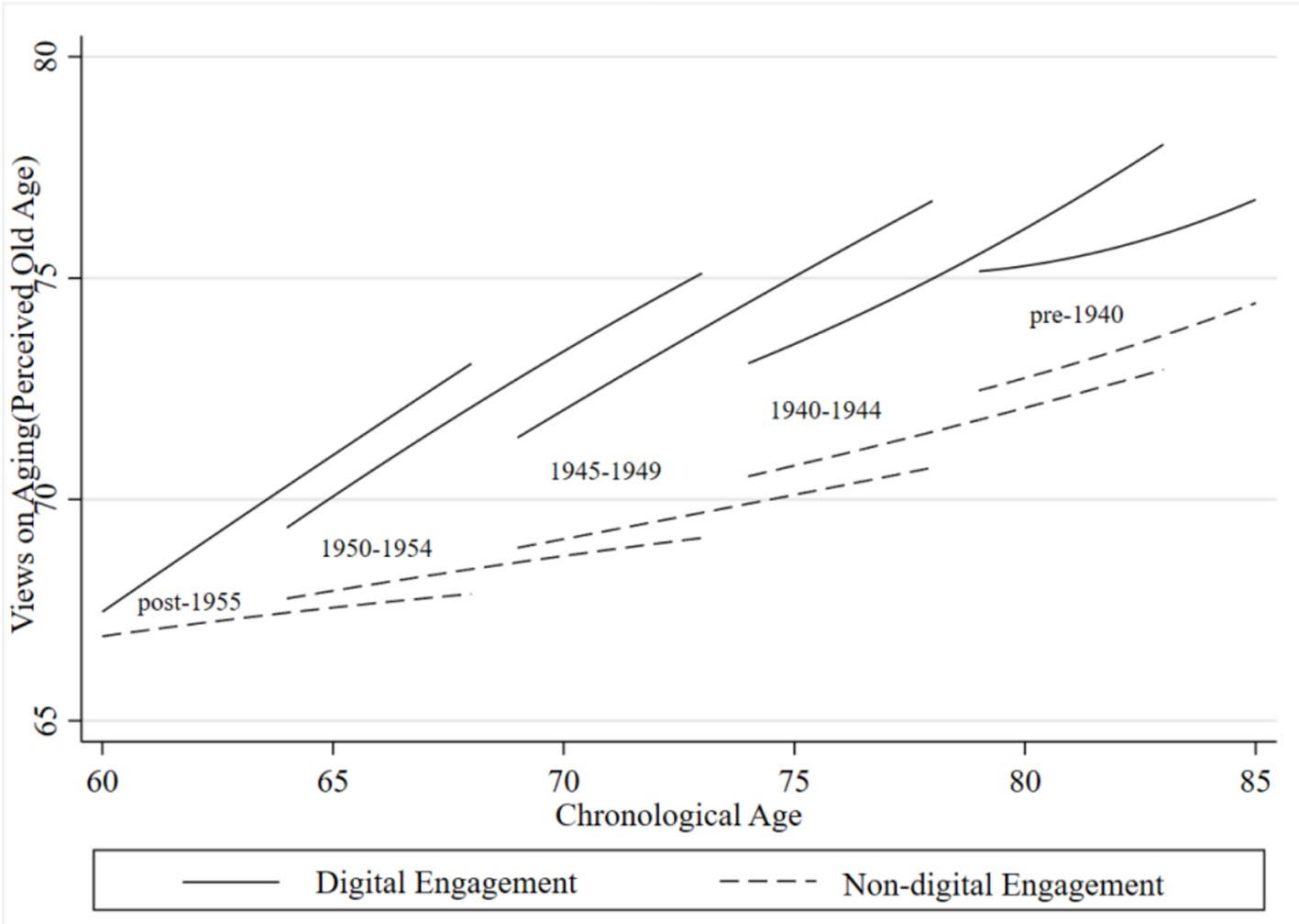
Age trajectories of perceived old age for older adults across generations. Compiled based on the 2018, 2020, and 2023 waves of CLASS data.

To further examine how VoA vary across cohorts and the role of digital engagement, we stratified the analytical sample into five cohorts based on birth year: pre-1940, 1940 ~ 1944, 1945 ~ 1949, 1950 ~ 1954, and post-1955. Figures 3, 4 display cohort-specific trajectories of VoA as a function of chronological age, derived from Model 4.

Figure 3 presents age trajectories of subjective age for older adults across generations stratified by digital engagement status. The results reveal substantial heterogeneity in digital engagement. Influences VoA across generations, characterized by a converging gap between digitally engaged and non-engaged groups with advancing chronological age. Overall, younger cohorts consistently reported lower subjective ages—indicating more positive VoA— at equivalent chronological ages compared to their older counterparts. However, a notable exception emerged among the pre-1945 cohort with digital engagement: their subjective age trajectories exhibited a pronounced upward slope with increasing chronological age, eventually exceeding those of their non-engaged peers at comparable ages. This reversal suggests that for this generation—socialized during an era of limited digitization—the cognitive demands associated with technology adaptation may have outweighed its psychological benefits. Rather than fostering positive self-perceptions, Digital engagement may have inadvertently intensified age-related anxiety through heightened awareness of the digital divide. Across most cohorts and age ranges, digitally engaged individuals maintained consistently lower subjective ages than their non-engaged counterparts, suggesting that digital engagement enhances positive self-perceptions through mechanisms such as improved information access, strengthened social connectivity, and expanded self-efficacy. Nevertheless, the divergence between groups progressively narrowed with advancing age, reflecting a convergence effect whereby subjective age in later life becomes increasingly constrained by age-related somatic factors, including health status and functional limitations, thereby weakening the beneficial effects of digital engagement.

Figure 4 illustrates age trajectories of perceived old age for older adults across generations stratified by digital engagement status. Across all groups, thresholds for defining old age rose progressively with chronological age, indicating increasingly positive VoA. Notably, the gap between digitally engaged and non-engaged groups exhibited a diverging pattern, widening rather than narrowing with advancing age. Among non-engaged older adults, a distinct cohort gradient emerged: younger generations set significantly lower thresholds for the onset of old age—indicating less positive perceptions—relative to older cohorts, who perceived old age as commencing at more advanced chronological ages. Conversely, among adults with digital engagement, this pattern was fully reversed. Younger cohorts with digital engagement reported substantially higher perceived old age for the onset of old age than older cohorts, suggesting that digital engagement amplifies positive VoA, particularly among younger generations. This pattern of generational differentiation plausibly reflects broader societal shifts—including rising life expectancy, educational expansion, and the dissemination of health-related knowledge—that have reshaped normative conceptions of aging, with digital engagement serving as a key conduit for these evolving standards. Notably, whereas the two groups exhibited convergence in VoA, they displayed pronounced divergence in perceived old age. This finding suggests that for aging perceptions grounded in normative judgments—rather than individualized self-assessments—digital engagement generates cumulative benefits across the life course, leading to increasingly divergent trajectories of VoA between digitally engaged and non-engaged older adults.

### 4.3 Robustness tests

**Figure 5:**
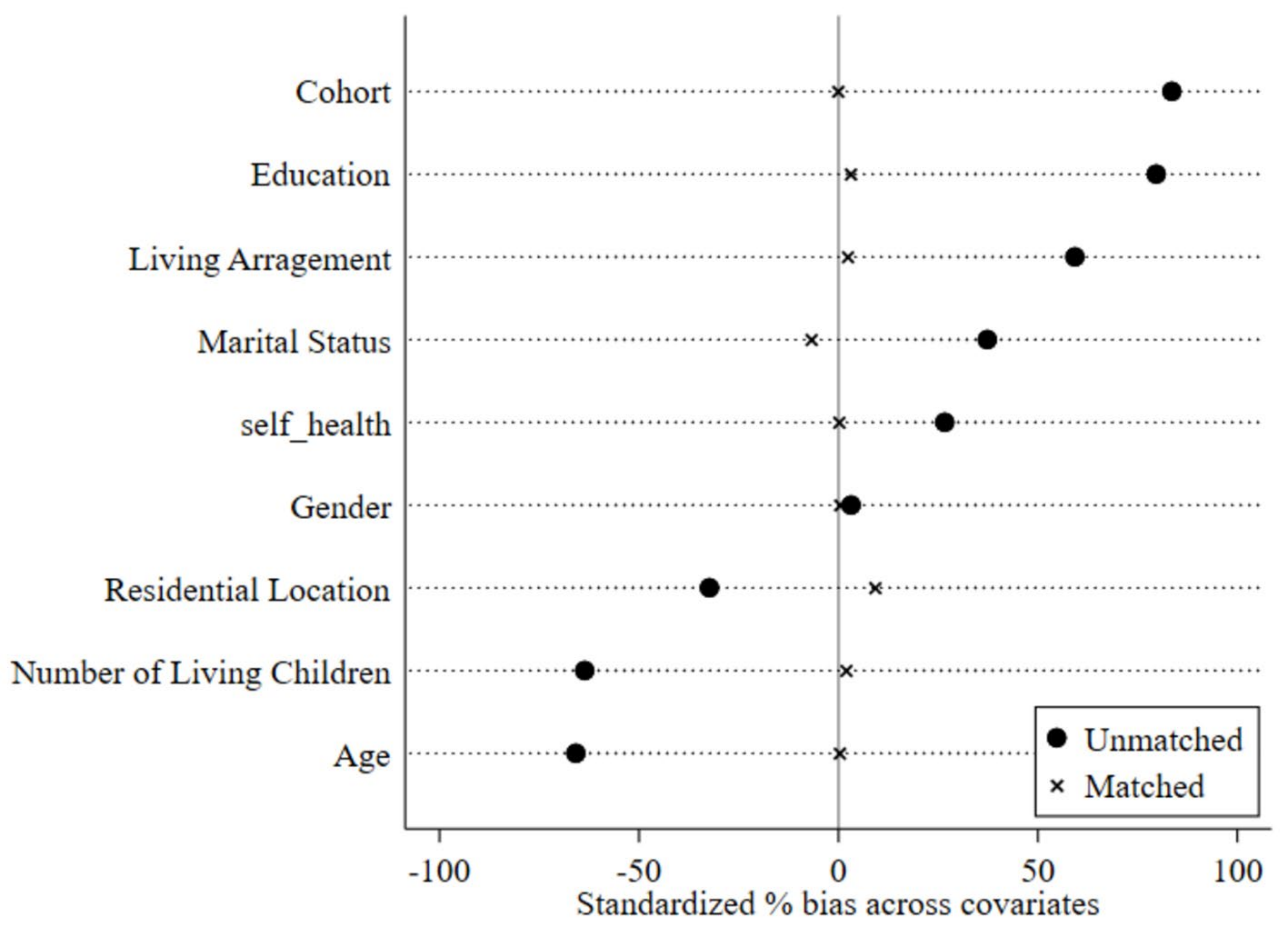
Balance test plot. Compiled based on the 2018, 2020, and 2023 waves of CLASS data.

To address potential selection bias, we conducted robustness checks using Propensity Score Matching. Individual-level covariates, including age, gender, and other demographic characteristics, were entered into a Probit regression to estimate propensity scores, followed by kernel matching using the Epanechnikov kernel function. Balance diagnostics are presented in Figure 5. Before matching, substantial standardized differences existed between the digitally engaged treatment group and the non-engaged matched group, with variables such as chronological age, birth cohort, and educational attainment exhibiting biases exceeding 50%. Following matching, all covariate imbalances were reduced to below 10%, indicating satisfactory covariate balance and comparable group composition. PSM validity tests are reported in Table 3. The Average Treatment effect on the Treated (ATT) reached statistical significance at the 1% level across all specifications, confirming that digital engagement significantly improves VoA even after controlling for observable confounders. Specifically, the matched treatment group exhibited a mean subjective age of −5.220 compared to −3.446 for the non-engaged matched group, yielding an ATT of −1.774 (*p* < 0.01), indicating that under comparable objective conditions, digitally engaged older adults perceive themselves as significantly younger than their non-engaged counterparts. Similarly, for perceived old age, the ATT of 2.612 (*p* < 0.01) demonstrates that individuals with digital engagement tended to set a later threshold for entering old age.

**Table 3:** Summary of average treatment effect results.

VoA	Treatment group	Control group	Average treatment effect on the treated	Standard error	*t*-value
Subjective age	−5.220	−3.446	−1.774^***^	0.338	−5.24
Perceived old age	71.918	69.305	2.612^***^	0.416	6.27

*p < 0.1; **p < 0.05; ***p < 0.01.

**Figure 6:**
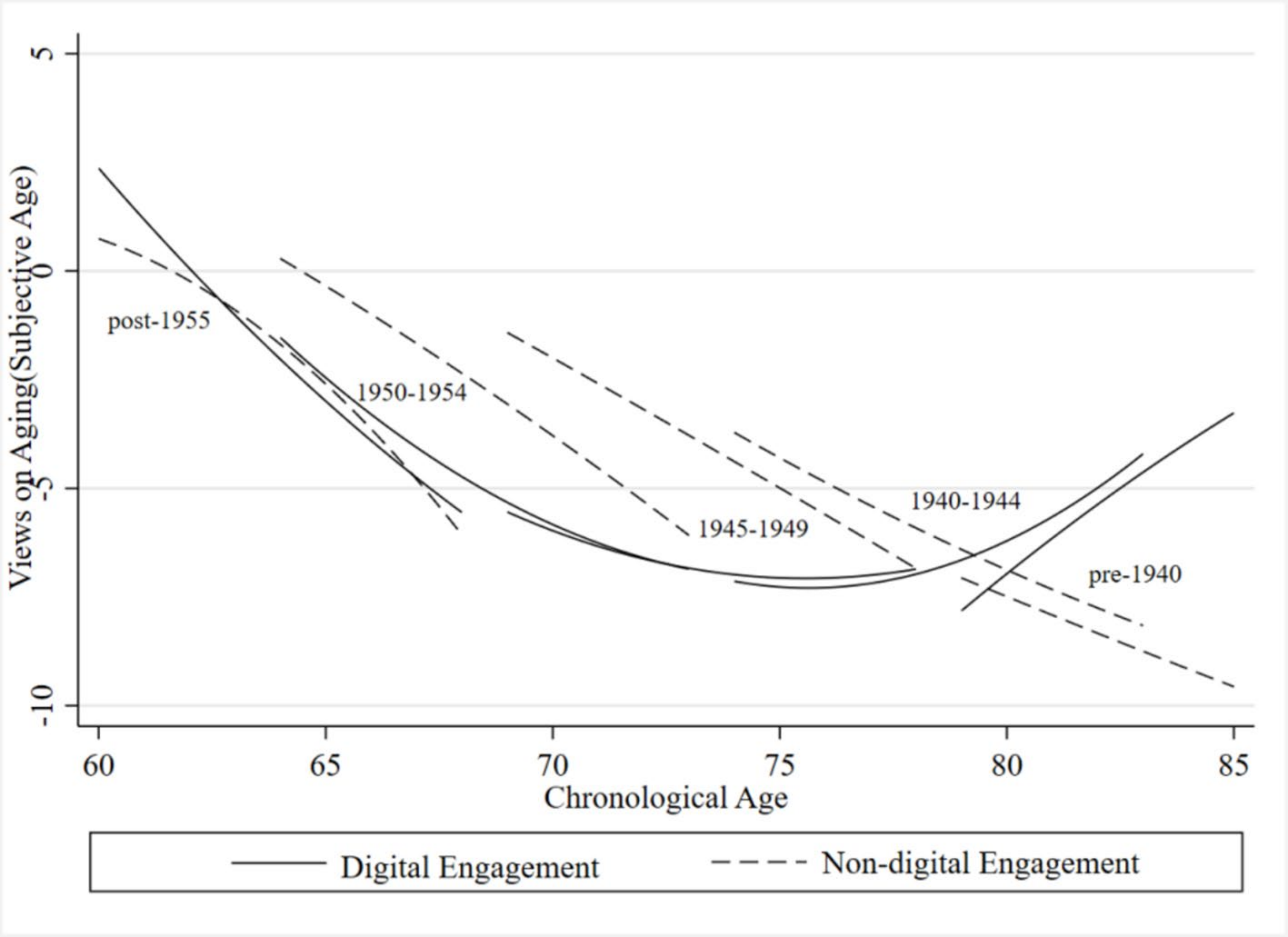
Age trajectories of subjective age for older adults across generations after matching. Compiled based on the 2018, 2020, and 2023 waves of CLASS data.

**Figure 7:**
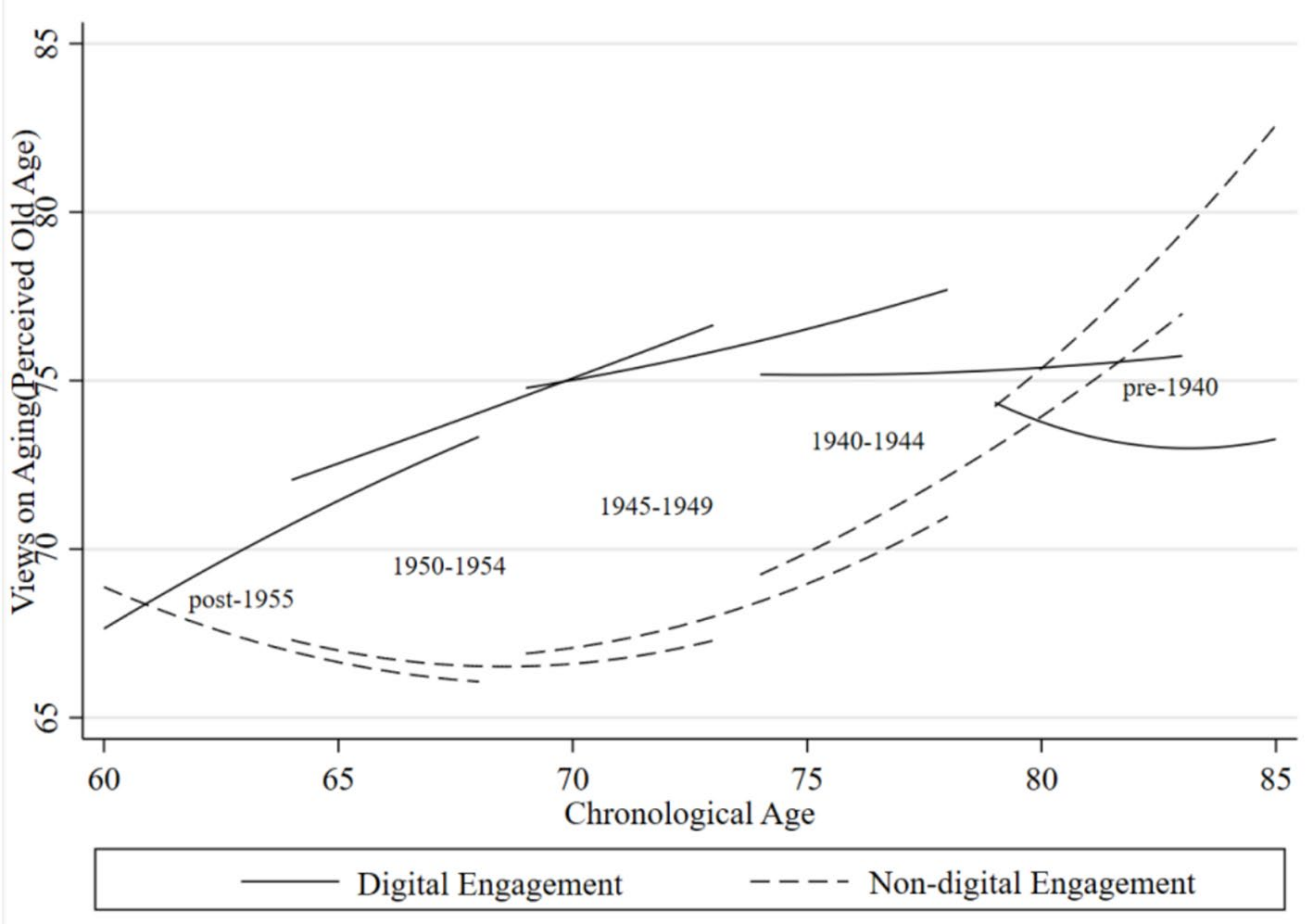
Age trajectories of perceived old age for older adults across generations after matching. Compiled based on the 2018, 2020, and 2023 waves of CLASS data.

Within the common support region of the matched sample, the growth curve model (Model 4) was re-estimated, incorporating matching weights. The age trajectories replotted from these re-estimated results (Figures 6, 7) remained broadly consistent with the patterns observed in the earlier figures, corroborating the significant effects of digital engagement across cohorts and life stages. Importantly, the trajectories for subjective age at advanced ages diverged from those descripted in the baseline regression section. In most cohorts, digitally engaged older adults entered advanced old age with VoA scores falling below (i.e., more negative than) those of their non-engaged peers, with the between-group gap exhibiting a widening rather than converging pattern. This suggests that the beneficial effects of digital engagement may weaken or reverse in the later life stages. This divergence likely stems in part from reduced sample sizes following matching. Additionally, it may reflect that the oldest-old face intensified functional constraints and digital strain when engaging with technology, compounded by declining health and contracting social networks—factors that collectively erode the psychological advantages of digital engagement among the very old, such that their subjective age perceptions may no longer maintain a comparative advantage.

## 5 Conclusion

Using data from the China Longitudinal Aging Social Survey in 2018, 2020, and 2023, this study employed growth curve modeling to examine age trajectories and generational differentiation in the effects of digital engagement on VoA, operationalized through both subjective age and perceived old age. The principal findings are as follows. First, VoA exhibit progressively positive trajectories across the life course. Subjective age declined with advancing chronological age, indicating that older adults develop increasingly younger self-perceptions, whereas perceived old age increased with chronological age, reflecting systematic postponement of the threshold defining entry into old age. These parallel trends collectively confirm consistent age-related improvements in VoA. Second, pronounced cohort differences characterizes older adults’ VoA. Later born cohorts consistently reported more favorable VoA—manifested as lower subjective ages and delayed perceptions of old age onset—attributable to their cumulative advantages in physiological health, educational attainment, and social resource access. These biological and social endowments jointly shape more positive cognitive aging profiles among newer generations. Third, digital engagement exerts significant positive influences on VoA. Across baseline regression specifications, cohort-stratified trajectory analyses, and propensity score matching robustness checks, digitally engaged older adults consistently reported younger subjective ages and postponed thresholds for perceived old age. Specifically, digital engagement significantly lowered subjective age, fostering younger self-perceptions, while elevating the chronological standard for defining old age, thereby delaying subjective entry into later life. These findings indicate that digital engagement provides tangible dividends for cognitive aging—not merely by expanding information access and social interaction opportunities, but also by enhancing intergenerational connectivity and self-efficacy, promoting more positive perceptions of aging. Fourth, the beneficial effects of digital engagement are stage-contingent. The promotive effects were most pronounced during the young-old phase (ages 60–75), attenuating upon entry into advanced old age and, in some cases, reversing with respect to subjective age. This pattern likely reflects the intensified technological barriers and psychological burdens confronting the oldest-old—including digital anxiety, information overload, and operational difficulties associated with physical decline—which collectively diminish the capacity of digital engagement to positively shape VoA at very advanced ages.

Based on these findings, we propose the following recommendations: First, strengthen top-level policy safeguards to establish the foundation for an age-friendly digital society. Integrate digital age-friendly adaptation into active aging strategies, coordinating comprehensive accessibility improvements across public and commercial services to bridge the digital divide. Develop protections for older adults’ digital rights and regulate cyberspace misconduct to create secure, welcoming digital environments, ensuring that digital engagement effectively enhances subjective age experiences and promotes positive aging. Second, enhance multi-dimensional cognitive guidance to cultivate scientific perspectives on aging. Embed positive aging concepts within eldercare services, community development, and media communications to dismantle stereotypes of “old age as decline.” Tailor communication strategies to cohort differences in VoA, fostering societal consensus on healthy aging across cohorts. Third, optimize digital service infrastructures to maximize the benefits of digital engagement. Advance age-friendly upgrades to digital services and implement tiered digital literacy training via community platforms, enabling older adults to expand information access and strengthen intergenerational connectivity. Regulate online information ecosystems to combat misinformation, ensuring that digital dividends fully support positive VoA. Fourth, construct differentiated, life-cycle service systems to precisely match diverse needs. For young-old adults, enrich integrated online-offline service provision to amplify the positive effects of digital engagement. For advanced-age older adults, reinforce offline safety-net provisions through a “digital assistance + offline support” hybrid model to overcome barriers to positive aging. Establish cross-departmental coordination mechanisms to consolidate resources and provide comprehensive support for active aging initiatives.

This study has several limitations. First, digital engagement is measured only as a binary variable, without distinguishing participation frequency, duration, intensity, or purpose. This prevents an in-depth analysis of its heterogeneous effects and undermines the robustness of causal identification. Future research should expand the dimensions of measurement and use more fine-grained data to capture the complex characteristics of digital participation. Second, although propensity score matching (PSM) is employed to account for differences in observable individual characteristics, the model may still be subject to bias from unobservable confounding factors, which imposes certain limitations on the interpretability of the results.

## Data Availability

Publicly available datasets were analyzed in this study. This data can be found at: http://jkzgyjy.ruc.edu.cn/sjzy/CLASSsjsq/index.htm.

## Footnotes

1 National Health Commission of the People’s Republic of China (NHC): https://www.nhc.gov.cn/.

2 《World Development Report 2016: Digital Dividends》https://www.worldbank.org/en/publication/wdr2016.
